# Macrophage IL-1**β** mediates atrial fibrillation risk in diabetic mice

**DOI:** 10.1172/jci.insight.171102

**Published:** 2024-06-18

**Authors:** Xiaoxu Zhou, Hong Liu, Feng Feng, Gyeoung-Jin Kang, Man Liu, Yugene Guo, Samuel C. Dudley

**Affiliations:** Division of Cardiology, Department of Medicine, Lillehei Heart Institute, University of Minnesota, Minneapolis, Minnesota, USA.

**Keywords:** Cardiology, Inflammation, Arrhythmias, Diabetes, Macrophages

## Abstract

Diabetes mellitus (DM) is an independent risk factor for atrial fibrillation (AF). The mechanisms underlying DM-associated AF are unclear. AF and DM are both related to inflammation. We investigated whether DM-associated inflammation contributed to AF risk. Mice were fed with high-fat diet to induce type II DM and were subjected to IL-1β antibodies, macrophage depletion by clodronate liposomes, a mitochondrial antioxidant (mitoTEMPO), or a cardiac ryanodine receptor 2 (RyR2) stabilizer (S107). All tests were performed at 36–38 weeks of age. DM mice presented with increased AF inducibility, enhanced mitochondrial reactive oxygen species (mitoROS) generation, and activated innate immunity in the atria, as evidenced by enhanced monocyte chemoattractant protein-1 (MCP-1) expression, macrophage infiltration, and IL-1β levels. Signs of aberrant RyR2 Ca^2+^ leak were observed in the atria of DM mice. IL-1β neutralization, macrophage depletion, and exposure to mitoTEMPO and S107 significantly ameliorated the AF vulnerability in DM mice. Atrial overexpression of MCP-1 increased AF occurrence in normal mice through the same mechanistic signaling cascade as observed in DM mice. In conclusion, macrophage-mediated IL-1β contributed to DM-associated AF risk through mitoROS modulation of RyR2 Ca^2+^ leak.

## Abstract 2

Diabetes mellitus–associated atrial fibrillation is caused by macrophage IL-1β through mitoROS modulation of RyR2 Ca^2+^ leak.

## Graphical abstract

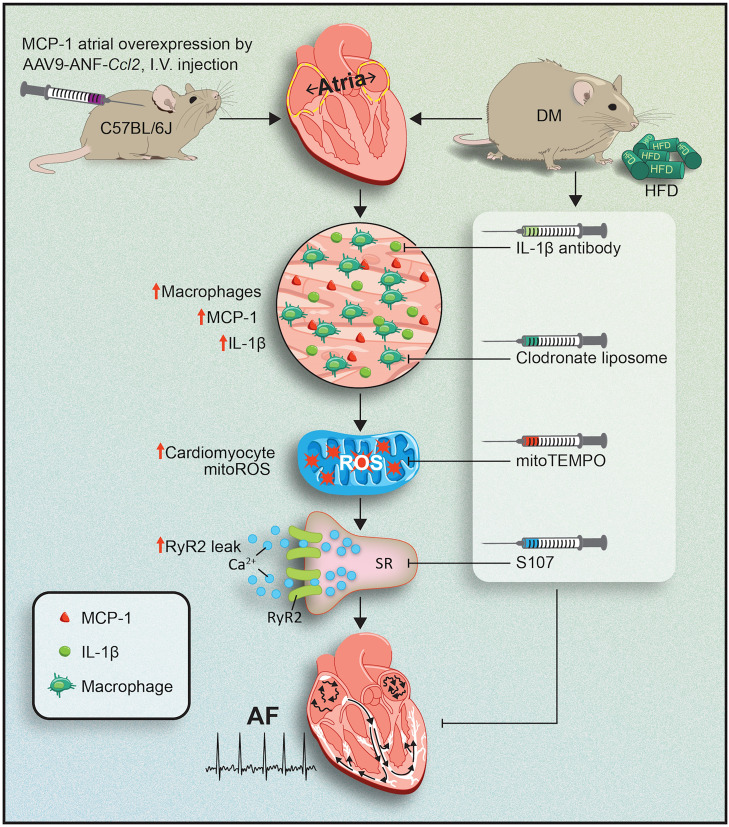

## Introduction

Type II diabetes mellitus (DM) is one of the most common chronic diseases in the world (1). DM is also an independent risk factor for the development of atrial fibrillation (AF) (2, 3), increasing the risk of developing AF by 40% (4). AF is the most prevalent human arrhythmia (5), and an estimated 2.5% of patients with AF have diabetes (4). The presence of comorbid DM and AF increases the risks of major cardiocerebrovascular events and all-cause mortality compared with either condition alone (6). DM causes electrical, structural, and autonomic remodeling in atria leading to the development of AF (7). Nevertheless, the exact cellular and molecular mechanisms inciting and maintaining DM-associated AF and diabetic atrial remodeling are not fully understood.

Recently, we have reported that DM activates inflammatory macrophages to secrete IL-1β, resulting in overproduction of mitochondrial reactive oxygen species (mitoROS) in the ventricle (8, 9). MitoROS contributes to DM-associated ventricular arrhythmic risk and heart failure with preserved ejection fraction (HFpEF) through oxidizing the downstream ryanodine receptor 2 (RyR2) channel and cardiac myosin binding protein C, respectively (9, 10). Considering the strong epidemiological interlink among DM, AF, and HFpEF, it is plausible that there may be common mechanistic signals underlying DM-associated cardiac electrical and functional abnormalities.

Therefore, in this study, we investigated whether macrophage-mediated IL-1β contributed to DM-associated AF via redox modulation of diastolic Ca^2+^ release.

## Results

### AF inducibility was increased in DM mice.

**Figure 1:**
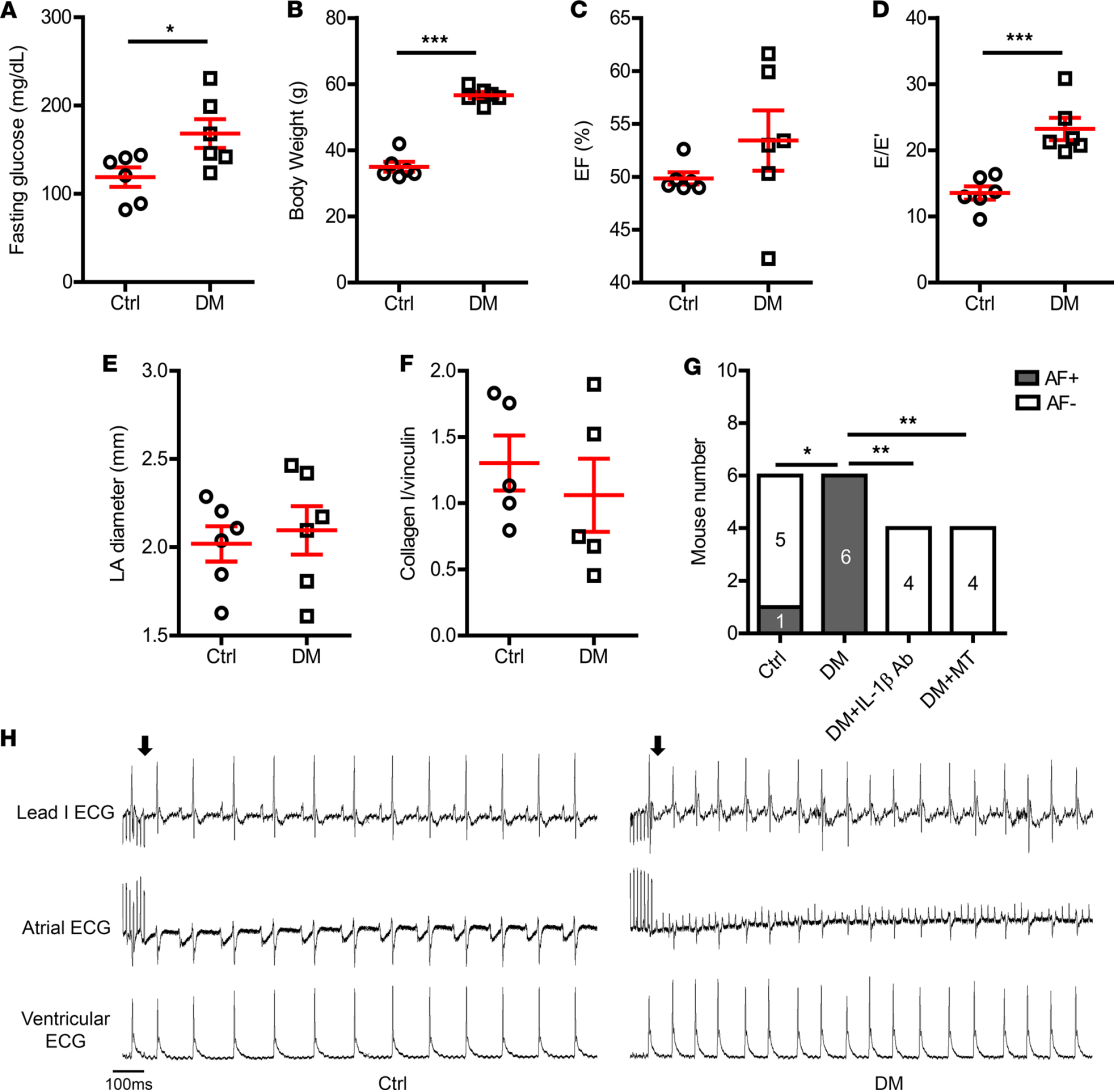
AF inducibility in DM mice. HFD-induced (**A**) high fasting glucose level and (**B**) obesity in mice. Echocardiographic evaluation indicated (**C**) preserved ejection fraction and (**D**) an increased ratio of transmitral Doppler early filling velocity to tissue Doppler early diastolic mitral annual velocity (E/E′). (**E**) Left atrial diameter by echocardiography and (**F**) atrial collagen I level by immunoblotting were comparable between control and DM mice. *N* = 5–6 mice per group. (**G**) DM mice had higher AF inducibility compared with the control mice; IL-1β–neutralizing antibody or mitochondrial antioxidant (mitoTEMPO) inhibited AF inducibility in DM mice. *N* is indicated within the bars. (**H**) Representative surface and intracardiac ECG traces showing induced AF in DM mice; arrows indicate the end of programmed stimulation. Scale bar: 100 ms. Data are shown as the mean ± SEM. Unpaired *t* test (**A**–**F**) or Fisher’s exact test (**G**) were used. *P < 0.05, **P < 0.01, ***P < 0.001. AF, atrial fibrillation; Ctrl, control; DM, diabetes mellitus; EF, ejection fraction; HFD, high-fat diet; LA, left atrium; MT, mitoTEMPO.

Type II DM was induced by feeding mice with high-fat diet (HFD) for at least 30 weeks. This HFD-induced DM model has been demonstrated and characterized in our previous studies (8, 10). Here, we recapitulated our previous observations that HFD induced hyperglycemia (Figure 1A, fasting glucose level, 119 ± 11 mg/dL in control vs. 168 ± 16 mg/dL in DM, *P* = 0.031) and obesity (Figure 1B, body weight, 35 ± 2 g in control vs. 57 ± 1 g in DM, *P* < 0.0001). Echocardiographic evaluation (Supplemental Table 1; supplemental material available online with this article; https://doi.org/10.1172/jci.insight.171102DS1) indicated impaired cardiac diastolic function in these HFD-induced DM mice (Figure 1D, ratio of transmitral Doppler early filling velocity to tissue Doppler early diastolic mitral annual velocity [E/E′], 13.6 ± 1.0 in control vs. 23.2 ± 1.7 in DM, *P* = 0.0006), whereas systolic function was preserved (Figure 1C, *P* = 0.246). The left atrial diameter (Figure 1E, *P* = 0.662) and the atrial collagen I level (Figure 1F, 1.30 ± 0.21 collagen I/vinculin in control vs. 1.06 ± 0.28 collagen I/vinculin in DM, *P* = 0.501; Supplemental Figure 1) were comparable between the control and DM mice. Programmed stimulation induced AF in 6 of 6 DM mice and only in 1 of 6 control mice (Figure 1, G and H, *P* = 0.015). These results suggested that AF vulnerability was increased in DM mice before apparent atrial structural remodeling was present.

### Innate immunity was activated in DM atria.

**Figure 2:**
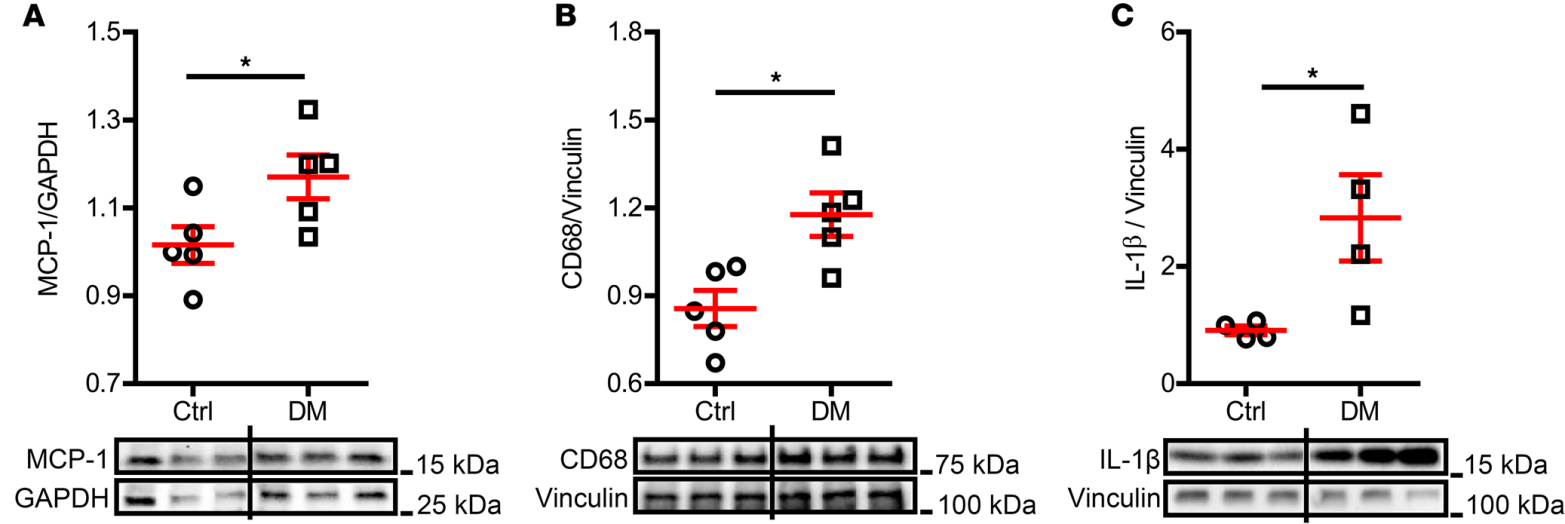
Inflammation was increased in DM atria. Expression level of (**A**) MCP-1, (**B**) macrophage marker CD68, and (**C**) inflammatory cytokine IL-1β were increased in DM atrial tissue. Representative immunoblotting images are shown. *N* = 4–5 mice per group. Data are shown as the mean ± SEM. Unpaired *t* test was used. *P < 0.05. Ctrl, control; DM, diabetes mellitus; MCP-1, monocyte chemoattractant protein-1.

To investigate whether innate immunity was activated in DM atria, we first compared the atrial level of monocyte chemoattractant protein-1 (MCP-1), a key chemokine that regulates macrophage migration and infiltration (11). As shown in Figure 2A, the atrial MCP-1 level was significantly higher in DM mice, compared with that of control mice (1.02 ± 0.04 MCP-1/GAPDH in control vs. 1.17 ± 0.05 MCP-1/GAPDH in DM, *P* = 0.045). An elevation of MCP-1 promotes macrophage infiltration (12). As expected, an enhanced expression of the macrophage marker CD68 was observed in DM mouse atria (Figure 2B, 0.86 ± 0.06 CD68/vinculin in control vs. 1.18 ± 0.07 CD68/vinculin in DM, *P* = 0.011), indicating increased macrophage infiltration in DM atria (Supplemental Figure 2). Activated macrophages are important sources of a potent inflammatory cytokine, IL-1β. In our study, we found that the IL-1β level was much higher in DM atria (Figure 2C, 2.83 ± 0.74 IL-1β/vinculin) than in control atria (0.91 ± 0.07 IL-1β/vinculin, *P* = 0.041). These results suggested that macrophage-mediated inflammation was enhanced in DM mouse atria.

### Macrophage-mediated IL-1β contributed to DM-associated AF.

**Figure 3:**
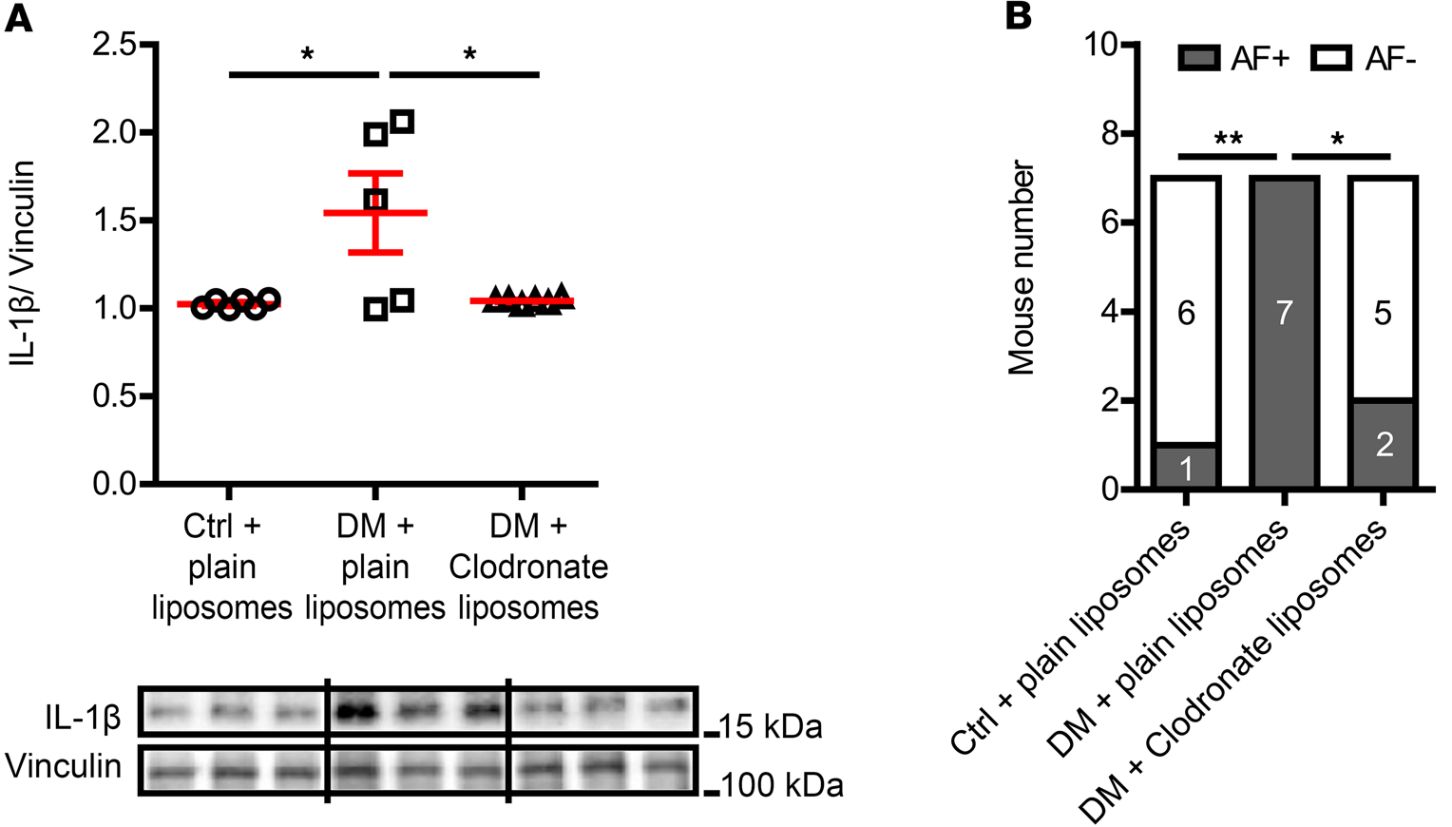
Macrophage-secreted IL-1β mediated DM-associated AF. (**A**) Atrial IL-1β level by immunoblotting was significantly lowered in macrophage depleted DM mice treated with clodronate liposomes compared with that in the plain liposome-treated DM mice. Representative immunoblotting images were shown. *N* = 5–6 mice per group. (**B**) Macrophage depletion by clodronate liposomes inhibited AF inducibility in DM mice. *N* is indicated within bars. Data are shown as the mean ± SEM. One-way ANOVA with Bonferroni’s post hoc tests (**A**) or Fisher’s exact test (**B**) were used. *P < 0.05, **P < 0.01. AF, atrial fibrillation; Ctrl, control; DM, diabetes mellitus.

We have previously reported that macrophage-mediated IL-1β contributed to DM-associated diastolic dysfunction (9). Diastolic dysfunction shares some common risk factors with AF, such as DM, hypertension, and obesity, etc. (13). To investigate whether a similar mechanism contributes to DM-associated AF, we treated DM mice with either clodronate liposomes or IL-1β–neutralizing antibodies for 2 weeks. We have demonstrated in a previous publication that a 2-week treatment with clodronate liposomes reduces the number of cardiac macrophages by 50% (9). In this study, we found that macrophage depletion remarkably reduced the IL-1β level in DM atria (Figure 3A, 1.54 ± 0.23 IL-1β/vinculin in DM + plain liposome vs. 1.04 ± 0.01 IL-1β/vinculin in DM + clodronate liposome, *P* = 0.037) to a level comparable to that in the control atria (1.03 ± 0.01 IL-1β/vinculin), suggesting that macrophages were a main source of IL-1β in DM atria. Both clodronate liposomes (Figure 3B, *P* = 0.021) and IL-1β antibodies (Figure 1G, *P* = 0.005) significantly mitigated the AF inducibility in DM mice. These findings indicated that IL-1β was the effector of macrophage-mediated inflammation in DM-associated AF.

### A mitochondria-targeted antioxidant eliminated AF risk in DM mice.

**Figure 4:**
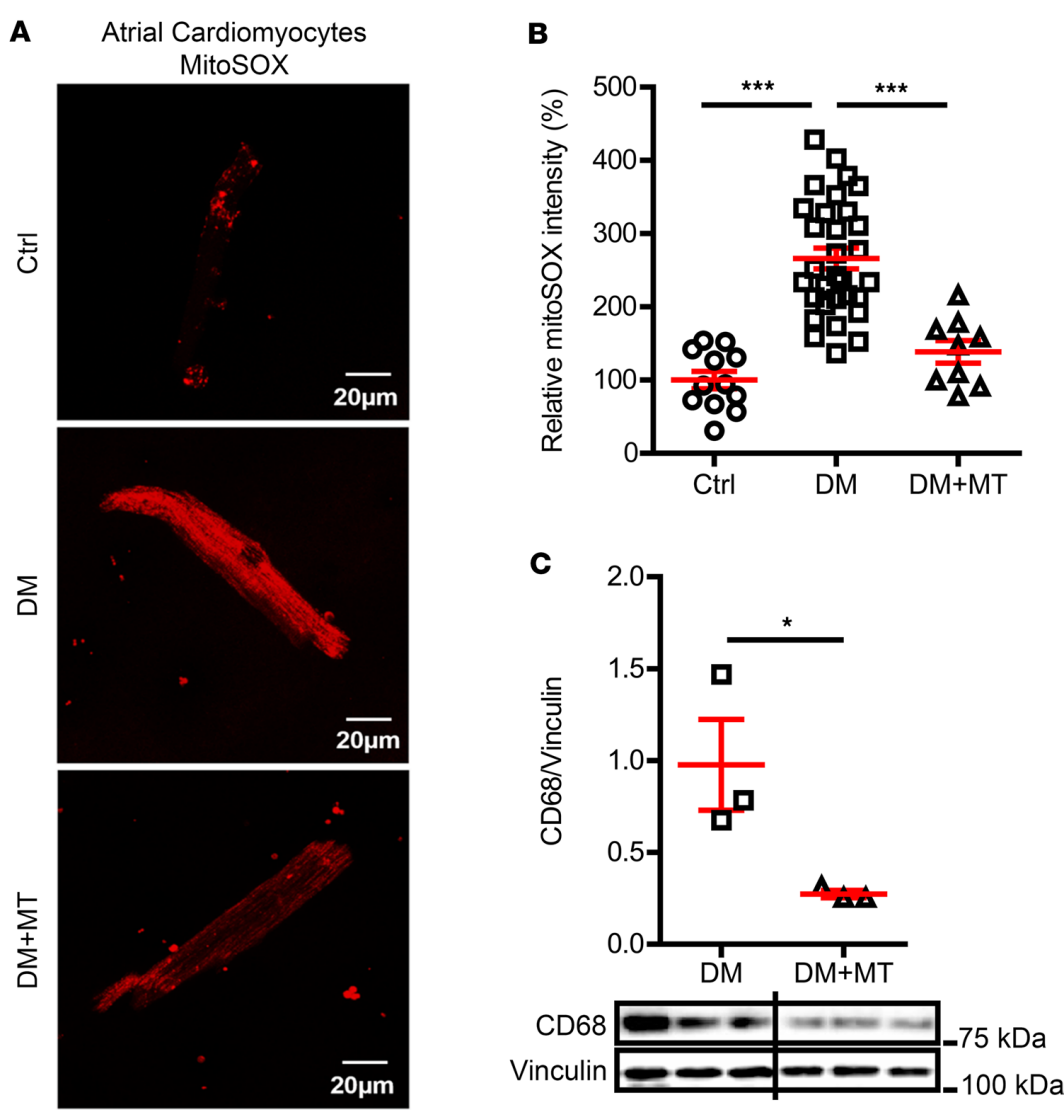
A mitochondrial antioxidant reduced AF inducibility. (**A**) Representative confocal microscopy images showing mitoROS in atrial cardiomyocytes by MitoSOX Red staining. Scale bar: 20 μm. (**B**) MitoSOX intensity was increased in DM atrial cardiomyocytes and reversed by mitochondrial antioxidant, mitoTEMPO. The atrial cardiomyocytes were isolated from 3 mice per group. (**C**) MitoTEMPO reduced the CD68 expression in DM atria. Representative immunoblotting images are shown. *N* = 3 mice per group. Data are shown as the mean ± SEM. One-way ANOVA with Bonferroni’s post hoc tests (**B**) or unpaired *t* test (**C**) were used. *P < 0.05, ***P < 0.001. Ctrl, control; DM, diabetes mellitus; MT, mitoTEMPO; mitoROS, mitochondrial reactive oxygen species.

AF is related to increased oxidative stress (14), and oxidative stress is considered a central mediator of AF (15). Mitochondria are the major ROS source in cardiomyocytes (CMs) (16) and mitoROS promotes AF (17). We have previously reported increased mitoROS levels in the ventricular CMs of DM mice and that IL-1β raises the mitoROS level of ventricular CMs in a dose-dependent response (8). Similar to that in DM ventricles, we found that the mitoROS level was more than doubled in DM atrial CMs (Figure 4, A and B, 100% ± 12% in control vs. 266% ± 14% in DM, *P* < 0.0001). A mitochondria-specific antioxidant, mitoTEMPO, was administered to DM mice for 2 weeks. At the end of the treatment, the atrial CM mitoROS level was significantly lowered (Figure 4, A and B, 138% ± 15%, *P* < 0.0001 vs. DM), and AF inducibility was completely inhibited by mitoTEMPO (Figure 1G, *P* = 0.005). In addition, mitoTEMPO reduced CD68 expression in DM atria (Figure 4C, 0.98 ± 0.25 CD68/vinculin in DM vs. 0.27 ± 0.02 CD68/vinculin in DM + mitoTEMPO, *P* = 0.048), suggesting reduced macrophage infiltration caused by inhibiting mitoROS. These data implied the involvement of atrial mitoROS in DM-associated AF risk and a positive feedback loop between mitoROS and atrial macrophage infiltration.

### Cardiac RyR2 Ca^2+^ leakage increased DM-associated AF risk.

**Figure 5:**
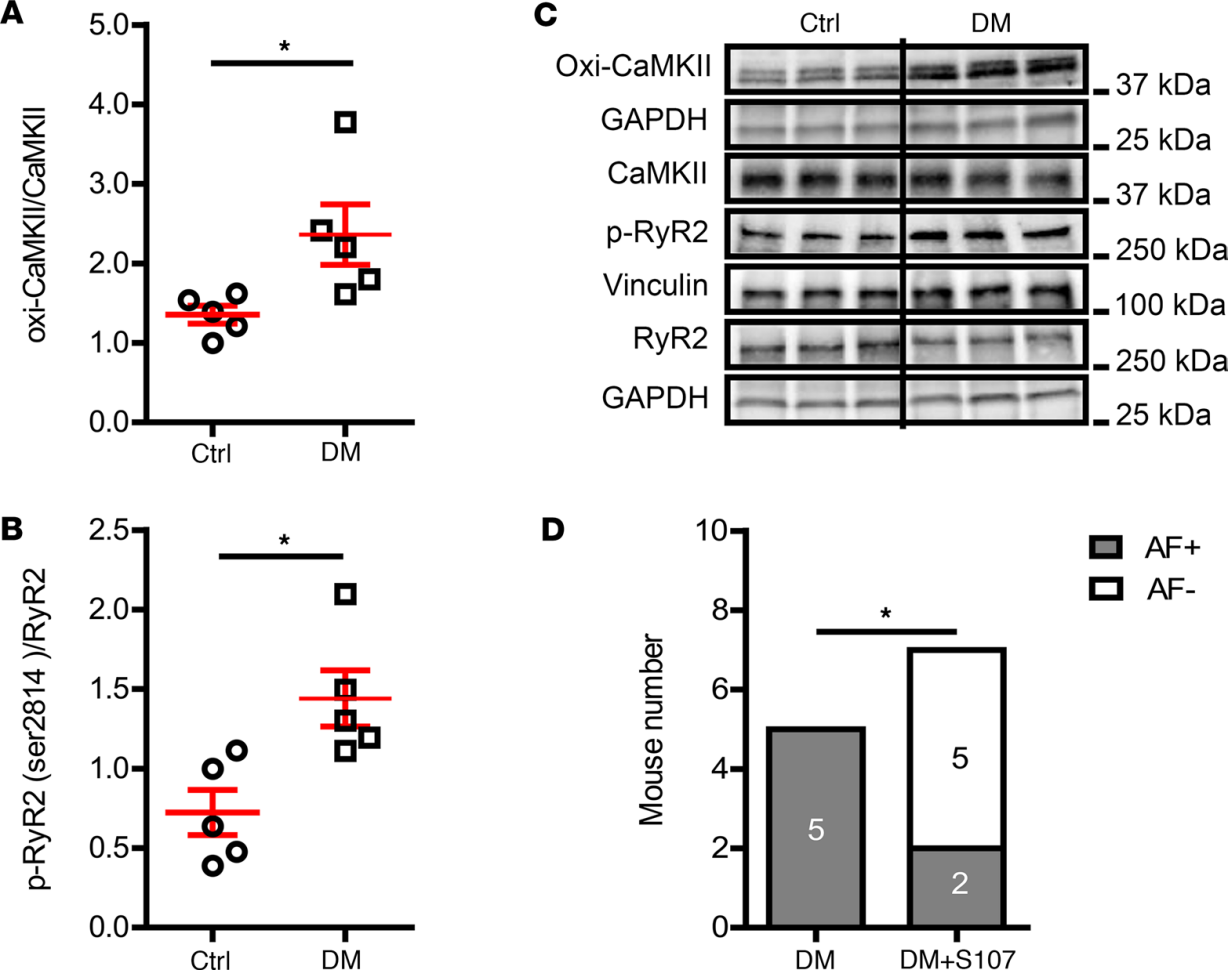
RyR2-mediated sarcoplasmic reticulum Ca^2+^ leak contributed to DM-associated AF. (**A**) Oxidized CaMKII and (**B**) phosphorylated RyR2 (Ser2814) levels determined by immunoblotting were increased in DM mouse atria. *N* = 5 mice per group. (**C**) Representative Western blot images of oxidized CaMKII, total CaMKII, p-RyR2-Ser2814, and total RyR2. (**D**) The RyR-calstabin interaction stabilizer S107 improved AF inducibility. *N* is indicated within bars. Data are shown as the mean ± SEM. Unpaired *t* test (**A** and **B**) or Fisher’s exact test (**D**) were used. *P < 0.05. AF, atrial fibrillation; oxi-CaMKII, oxidized Ca^2+^/calmodulin-dependent protein kinase II; Ctrl, control; DM, diabetes mellitus; RyR2, ryanodine receptor 2.

AF is associated with increased Ca^2+^ release from sarcoplasmic reticulum (SR), and RyR2 is the major Ca^2+^ release channel in the SR of CMs (18, 19). Phosphorylation of RyR2 at Ser2814 by activated Ca^2+^/calmodulin-dependent protein kinase II (CaMKII) increases SR Ca^2+^ leak in human AF (20). MitoROS activates CaMKII by oxidation with consequent RyR2 (Ser2814) phosphorylation (21). We therefore tested whether CaMKII oxidation-mediated RyR2 Ca^2+^ leakage was involved in DM-associated AF. In DM mice, the atrial expression of oxidized CaMKII (oxi-CaMKII) (Figure 5, A and C, 1.35 ± 0.11 oxi-CaMKII/CaMKII in control vs. 2.36 ± 0.38 oxi-CaMKII/CaMKII in DM, *P* = 0.035) as well as phosphorylated RyR2 (Ser2814) (Figure 5, B and C, 0.72 ± 0.14 p-RyR2 (Ser2814)/RyR2 in control vs. 1.44 ± 0.18 p-RyR2 (Ser2814)/RyR2 in DM, *P* = 0.013) were increased when compared with those of the control mice. More importantly, treating mice with S107, a RyR-calstabin interaction stabilizer, to reduce SR Ca^2+^ leak, significantly improved AF inducibility in DM mice (Figure 5D, *P* = 0.028). These data indicated that RyR2 Ca^2+^ leak, possibly mediated by phosphorylation via oxi-CaMKII, contributed to the increased AF vulnerability in DM.

### Atrial MCP-1 overexpression led to AF in normal mice via the same signaling cascade.

**Figure 6:**
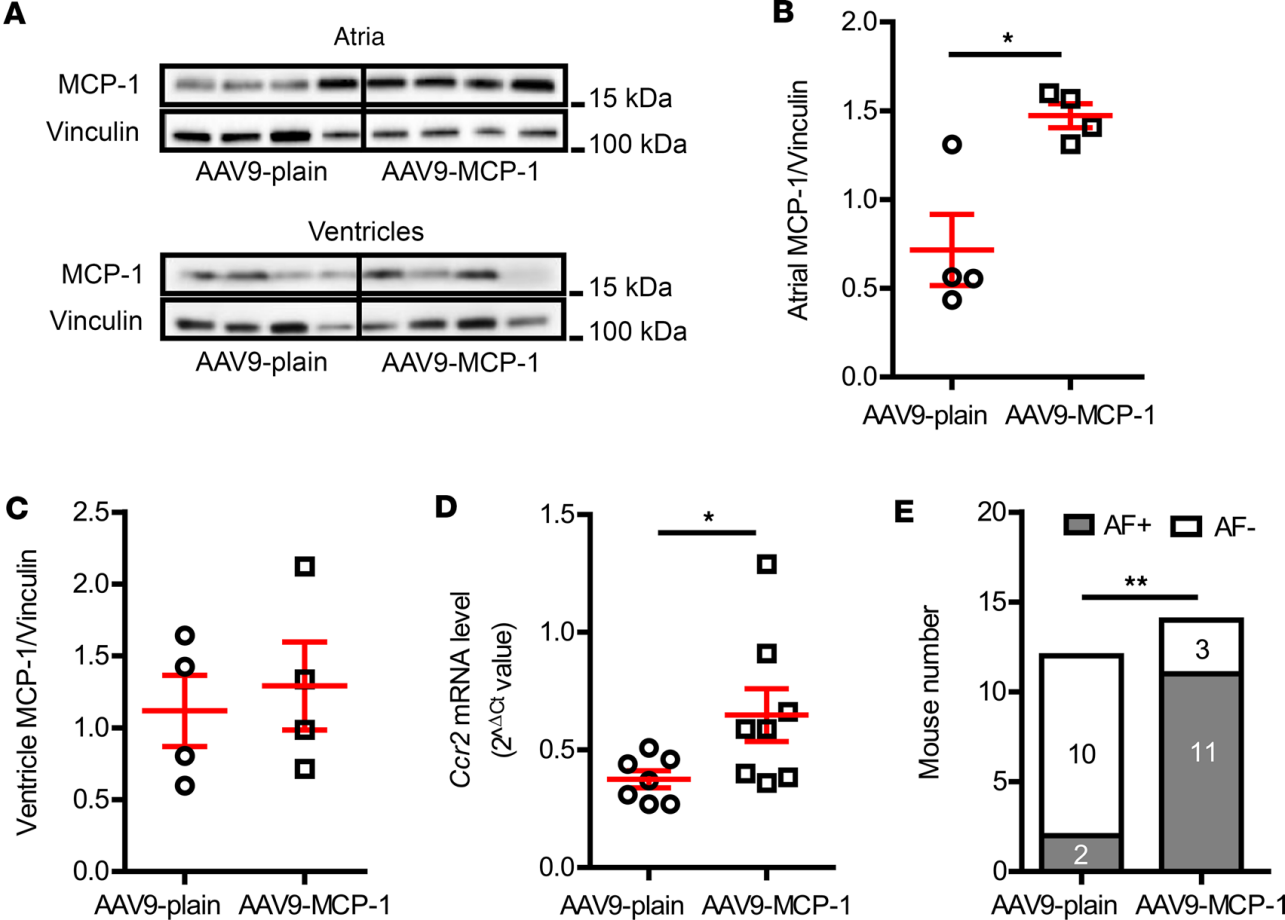
Atrial MCP-1 overexpression increased AF risk in normal mice. (**A**) Representative immunoblotting images of MCP-1 expression in mouse atria and ventricles 1 month after AAV9 vector injection. (**B**) Atrial but not (**C**) ventricular MCP-1 levels were elevated in the mice receiving AAV9-ANF-MCP-1 vector injection. (**D**) Atrial *Ccr2* mRNA level was increased in the MCP-1–overexpressing mice. *N* = 7–8 mice per group. (**E**) MCP-1 atrial overexpressing mice exhibited high AF inducibility. *N* is indicated within the bars. Data are shown as the mean ± SEM. Unpaired *t* test (**B**–**D**) or Fisher’s exact test (**E**) were used. *P < 0.05, **P < 0.01. AAV9, adeno-associated virus serotype 9; AF, atrial fibrillation; ANF, atrial natriuretic factor; MCP-1, monocyte chemoattractant protein-1.

To further confirm the association between macrophages and AF, atria-specific adeno-associated viral vectors (serotype 9 [AAV9] with the promoter of atrial natriuretic factor) were intravenously administered into otherwise normal mice. As shown in Figure 6, A and B, MCP-1 levels were specifically upregulated in the atria CMs (0.72 ± 0.20 atrial MCP-1/vinculin in AAV9-plain vs. 1.47 ± 0.07 atrial MCP-1/vinculin in AAV9-MCP-1, *P* = 0.012) without a change in the ventricles (Figure 6, A and C, 1.12 ± 0.25 ventricle MCP-1/vinculin in AAV9-plain vs. 1.29 ± 0.31 ventricle MCP-1/vinculin in AAV9-MCP-1, *P* = 0.676). MCP-1 attracts macrophages mostly through the interaction with their receptor CCR2 (11), and previously, we have reported that MCP-1 and CCR2^+^ macrophages are increased in DM hearts (9). Consistent with this previous result, we found that *Ccr2* gene expression was significantly enhanced in MCP-1–overexpressing atria (Figure 6D, 0.38 ± 0.04 2^–ΔCt^ in AAV9-plain vs. 0.65 ± 0.11 2^–ΔCt^ in AAV9-MCP-1, *P* = 0.048), implying increased CCR2^+^ macrophage infiltration. Concurrently, atrial MCP-1 overexpression (OE) mice were more prone to inducible AF. As shown in Figure 6E, 11 of 14 MCP-1 OE mice were AF inducible compared with only 2 of 12 control mice injected with AAV9 plain vector (*P* = 0.005). These results indicated that atrial MCP-1 OE increased AF vulnerability, presumably via inducing macrophage infiltration.

**Figure 7:**
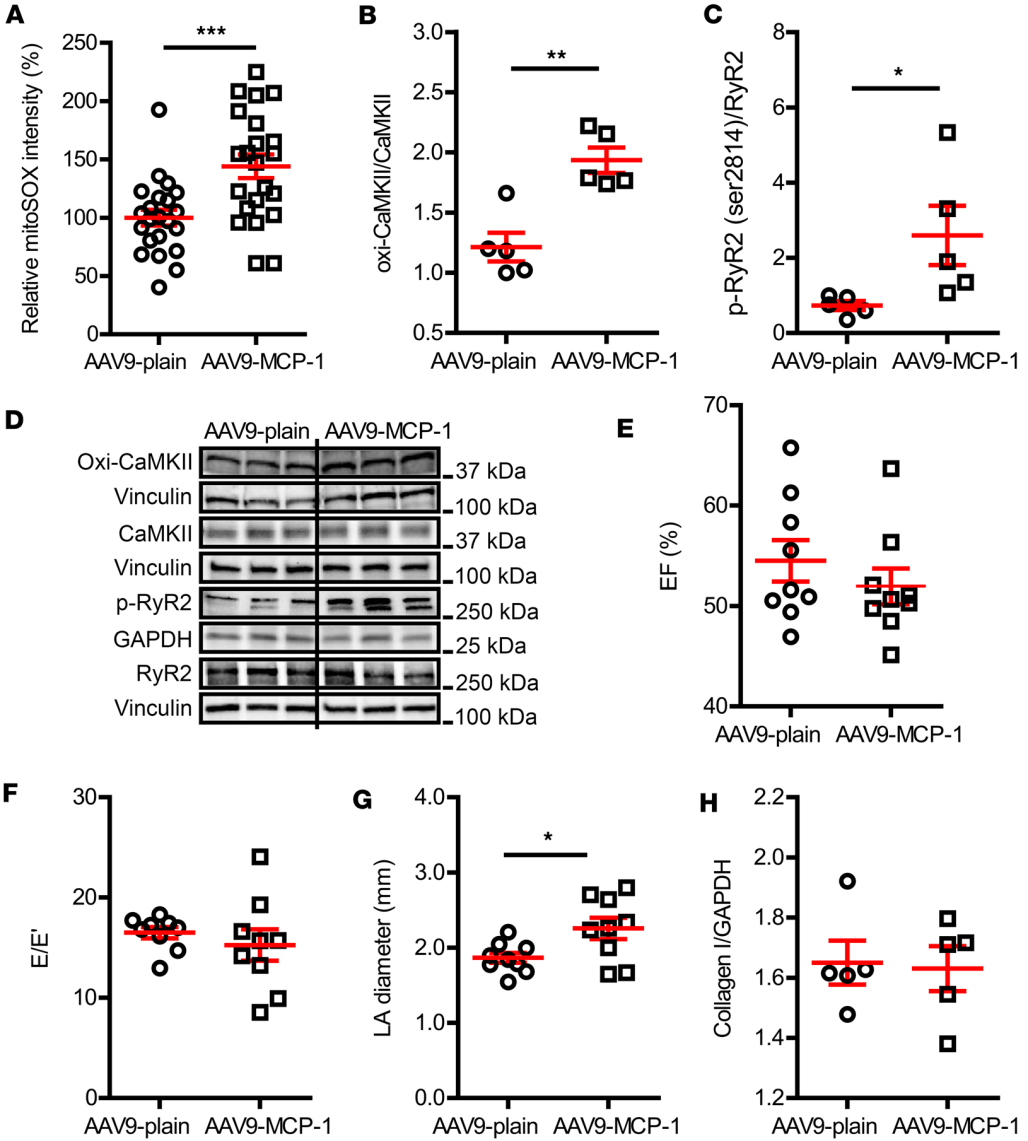
Cardiac characterization of atrial MCP-1–overexpressing mice. (**A**) MitoSOX intensity was enhanced in the atrial cardiomyocytes overexpressing MCP-1. The atrial cardiomyocytes were isolated from 2 mice per group. (**B**) Oxi-CaMKII and (**C**) p-RyR2 (Ser2814) levels were increased in MCP-1–overexpressing atria. (**D**) Representative immunoblotting images of oxidized CaMKII, total CaMKII, p-RyR2-Ser2814, and total RyR2. (**E**–**G**) Echocardiographic evaluation of EF, E/E′, and left atrial diameter, respectively. (**H**) Atrial collagen I level in AAV9-plain and AAV9-MCP-1 vector–treated mice. *N* = 4–9 mice per group. Data are shown as the mean ± SEM. Unpaired *t* test (**A**–**C** and **E**–**H**) was used. *P < 0.05, **P < 0.01, ***P < 0.001. AAV9, adeno-associated virus serotype 9; AF, atrial fibrillation; E/E′, ratio of transmitral Doppler early filling velocity to tissue Doppler early diastolic mitral annual velocity; EF, ejection fraction; LA, left atrium; MCP-1, monocyte chemoattractant protein-1; oxi-CaMKII, oxidized Ca^2+^/calmodulin-dependent protein kinase II; RyR2, ryanodine receptor 2.

Moreover, a similar signaling cascade as observed in DM atria was present in MCP-1 OE mice. The mitoROS level was elevated by 1.5-fold in atrial CMs from MCP-1 OE mice (Figure 7A, *P* = 0.0008) compared with that in the AAV9-plain vector–treated mice. Both oxi-CaMKII and phosphorylated RyR2 (Ser2814) were significantly higher in MCP-1 OE atria than in control atria. The oxi-CaMKII increased from 1.21 ± 0.12 oxi-CaMKII/CaMKII in control mice to 1.94 ± 0.10 oxi-CaMKII/CaMKII in MCP-1 OE mice (Figure 7, B and D, *P* = 0.002), and p-RyR2 (Ser2814) increased from 0.73 ± 0.12 p-RyR2 (Ser2814)/RyR2 in control mice to 2.60 ± 0.79 p-RyR2 (Ser2814)/RyR2 in MCP-1 OE mice (Figure 7, C and D, *P* = 0.047). These data suggested that, similar to DM-associated AF, mitoROS-mediated RyR2 modification linked the inflammation and AF in MCP-1 OE mice.

In contrast to DM mice where AF risk may have been encouraged by cardiac diastolic dysfunction, both cardiac systolic (Figure 7E, ejection fraction, 55% ± 2% in control mice vs. 52 ± 2% in MCP-1 OE mice, *P* = 0.366) and diastolic function (Figure 7F, E/E′, 16.5 ± 0.6 in control vs. 15.3 ± 1.6 in MCP-1 OE, *P* = 0.467) were preserved in MCP-1 OE mice (Supplemental Table 2). Although the left atrium was slightly enlarged (Figure 7G, 1.9 ± 0.1 mm in control vs. 2.3 ± 0.1 mm in MCP-OE mice, *P* = 0.023), atrial collagen level was not altered by MCP-1 OE (Figure 7H, 1.7 ± 0.1 collagen I/GAPDH in control mice vs. 1.6 ± 0.1 collagen I/GAPDH in MCP-1 OE mice, *P* = 0.853). These results indicated that inflammation-mediated AF risk was independent of cardiac function.

## Discussion

In this study, we found that DM caused AF vulnerability accompanied by enhanced atrial MCP-1 and IL-1β levels, increased atrial macrophage infiltration, and elevated mitoROS production and RyR2 phosphorylation in atrial CMs. Neutralizing IL-1β, depleting macrophages, scavenging mitoROS, or blocking Ca^2+^ leak from RyR2 channels improved AF vulnerability in DM mice. The above signaling cascade was further confirmed in the atrial specific MCP-1–overexpressing mice in which AF risk was significantly enhanced. Taken together, these findings indicated that activated innate immunity contributed to the DM-associated AF tendency via IL-1β–mediated atrial electrical remodeling of RyR2 through mitoROS modulation.

Both DM and AF are associated with inflammation (22–25). DM is well known as a chronic inflammatory disease. Activation of the innate immune response is closely involved in the pathogenesis of type II DM (26). Macrophages are a major component of innate immunity and the major immune cell population in hearts (27, 28). Patients with type II DM present with higher plasma MCP-1 levels and increased CD68^+^ macrophages in the atrial myocardium when compared with patients without DM (29, 30). Macrophages can adopt proinflammatory or antiinflammatory phenotype, and DM favors the proinflammatory macrophages (31).

There is considerable evidence to suggest that macrophages can contribute to AF (32). The main population of immune cells in human left atrial appendages of patients with AF are active monocytes/macrophages (27, 33). Increased MCP-1 and proinflammatory macrophage infiltration in atria have been reported in both humans and animals with AF (33–39). Blocking monocyte recruitment reduces atrial macrophage infiltration and lowers the incidence of hypertension-induced AF (40). Increased macrophage proinflammatory polarization (Inos^+^ and Arg1^–^) is found in the mouse and canine atria after LPS was used to induce AF (41). We and others have reported a similar role of macrophages on the risk of diabetic ventricular arrhythmic (8, 42). Therefore, macrophage-mediated inflammation may be a key link between DM and arrhythmia.

In the current study, we found enhanced MCP-1 and macrophage infiltration in the DM mouse atria (Figure 2, A and B, and Supplemental Figure 2). The increased IL-1β in diabetic atria (Figure 2C) and the upregulated gene expression of CCR2, a proinflammatory macrophage marker, in MCP-1–overexpressing atria (Figure 6D) support that the accumulated macrophages in DM atria were proinflammatory. That is consistent with our previous report of an increased shift toward proinflammatory macrophages in the ventricles of the same DM mouse model (9). Moreover, we observed that depleting macrophages attenuated DM-associated AF vulnerability (Figure 3B), and atrial specific overexpression of MCP-1 induced AF in normal mice (Figure 6E), suggesting the central contribution of proinflammatory macrophages in DM-associated AF.

Although macrophage depletion was sufficient to reduce arrhythmic risk, we cannot rule out a role for other inflammatory cell types in DM-associated AF. In another report, neutrophils are a main ROS source and play a profibrotic role in AF genesis, and T cells and B cells contribute to AF via regulating innate immunity and producing autoantibodies, respectively (27). Those 3 leukocyte subsets have been found to be involved in diabetic cardiomyopathy (31, 43).

Proinflammatory macrophages secrete inflammatory cytokines, such as IL-1β, IL-6, and TNF-α, all of which are elevated in patients with AF or associated with the outcome of AF (44–49). Elevated IL-1β level is an independent risk factor for persistent AF in patients after coronary artery bypass grafting surgery (45). AF is remarkably associated with elevated IL-6 in the patients with coronary artery disease, chronic obstructive pulmonary disease, chronic kidney diseases, and many other systemic inflammatory diseases (49–51). High levels of TNF-α are reported in patients with valvular AF (52). In LPS-induced AF, proinflammatory macrophages induce atrial electrical remodeling through IL-1β and TNF-α (41). In our DM mouse model, we showed that DM-associated AF was at least partially mediated by macrophage-secreted IL-1β, as evidenced by the efficacy of macrophage depletion and IL-1β neutralization in blocking AF (Figure 1G and Figure 3B). Nevertheless, we did not rule out the role of other cytokines in DM-associated AF. Furthermore, we did not examine other cell sources of IL-1β. For example, CMs can also release inflammatory cytokines through the activation of the NLRP3 (NACHT, LRR, and PYD domain containing protein 3) inflammasome that may contribute to the proclivity for AF (53). Nevertheless, in the present study, macrophage depletion normalized atrial IL-1β levels (Figure 3A), establishing macrophages as the main source of IL-1β in DM atria.

Furthermore, we investigated the mechanism whereby IL-1β causes AF in DM. IL-1β is known to activate mitoROS (54–56). In obesity and DM, oxidative stress is a central mediator of AF (15). In atrial tissue, increased mitoROS is observed in patients with AF and animal models of AF (17, 57, 58). MitoROS can cause AF through promotion of SR Ca^2+^ leak via RyR2 oxidation or CaMKII-mediated phosphorylation (17, 59, 60). CaMKII can be activated by mitoROS via oxidation (15, 61, 62), and oxi-CaMKII is elevated in AF atria (63). We have similar observations that DM elevated mitoROS production and CaMKII oxidation in atria and led to increased RyR2 phosphorylation (Figure 4 and Figure 5). A mitochondrial antioxidant protected DM mice from inducible AF (Figure 1G), providing additional proof of a redox mechanism in DM-associated AF. In addition to redox modulation of intracellular Ca^2+^ homeostasis, mitoROS can also promote AF through perpetuating inflammation via activating the NLRP3 inflammasome and driving release of inflammatory cytokines, such as IL-1β and IL-18 (64). This is reflected in our finding that scavenging mitoROS inhibited macrophage infiltration (Figure 4C), which is proof of a vicious positive feedback loop between mitoROS and macrophage-mediated inflammation in the development of DM-associated AF.

The suppression of DM-associated AF risk by RyR2 stabilization (S107) indicated the contribution of RyR2 channels to AF in DM, potentially by oxi-CaMKII–mediated phosphorylation (Figure 5). Similarly, Mesubi et al. reported that diabetic AF is dependent on oxi-CaMKII–activated RyR2 Ca^2+^ leak (65). Nevertheless, we cannot rule out the possibility of direct RyR2 oxidation by ROS leading to the pathogenesis of AF, as reported by Xie et al. (17).

IL-1β can also contribute to AF by affecting other cardiac ion channels or atrial fibrosis (41, 46, 66). In LPS-induced AF, IL-1β contributes to the atrial electrical remodeling by leading to downregulated L-type calcium channel currents and decreased atrial effective refractory period (41). Inhibiting IL-1β–induced atrial fibrosis prevents postoperative AF (66). In our DM mouse model, atrial fibrosis was not a potent contributor to AF because atrial collagen level was not altered (Figure 1F and Supplemental Figure 1). Instead, our data suggest RyR2-mediated Ca^2+^ leak as a potential trigger mechanism for AF initiation (Figure 5). Nevertheless, the roles of other types of electrical remodeling in DM-associated AF were not examined and need further clarification.

Previously, we have investigated the inflammatory mechanism in DM-associated ventricular arrhythmic risk and diastolic dysfunction (8, 9). In the present study, we found that DM-associated AF shared a common mechanistic signaling cascade with diabetic diastolic dysfunction as well as ventricular arrhythmic risk, namely MCP-1 elevation, macrophage infiltration, IL-1β secretion, mitoROS overproduction, and posttranslational modification of target proteins. This finding is consistent with the epidemiological association of AF and diastolic heart failure (67). A recent randomized double-blind placebo-controlled clinical trial (the CANTOS trial) showed that antiinflammatory therapy with an IL-1β–specific antibody (canakinumab) substantially reduced cardiovascular events (68), supporting our conclusion that IL-1β is a key signaling component in DM-associated cardiovascular complications.

It must be recognized that mice may not be an ideal model of human electrophysiological diseases. Mouse cardiac physiology differs from that of humans in aspects such as heart size, basal heart rate, action potential duration, and ionic currents for CM repolarization (69). Thus, extrapolating mouse data for human electrophysiological implications needs to be done cautiously.

In summary, DM results in activation of a cardiac innate immune response associated with increased AF risk. AF vulnerability could be ameliorated by depleting macrophages, antagonizing IL-1β, scavenging mitoROS, or inhibiting SR Ca^2+^ leak. Each of these approaches represents a possible new therapy for preventing DM-associated AF risk.

## Methods

### Sex as a biological variable.

Our study only examined male diabetic mice, as female C57BL/6J mice were less susceptible to HFD-induced DM (70). For mice overexpressing MCP-1 in atria in this study, both male and female mice were used, and similar findings were observed in both sexes.

### Animal experimental protocol.

DM was induced by feeding male C57BL/6J mice (The Jackson Laboratory) with HFD (60 kcal% fat, Research Diet) starting at 6 weeks of age, and DM was confirmed by fasting blood glucose level using a glucometer (ACCU-CHEK, Roche Applied Science). Sex- and age-matched C57BL/6J mice, fed with regular chow (Harlan), were used as controls. At the age of 34–36 weeks, all DM mice were randomly assigned to one of the following 4 treatments: (a) intraperitoneal injection of 300 μg IL-1β–neutralizing antibodies (BioLegend) every 3 days for 2 weeks to suppress IL-1β; (b) a bolus intravenous injection of 0.25 mL clodronate liposomes (reconstituted with 4.7 mL sterile water, FormuMax Scientific Inc.), followed by intraperitoneal injection of 0.3 mL clodronate liposomes biweekly for 2 weeks to deplete macrophages; (c) daily intraperitoneal injection of 1 mg/kg mitoTEMPO (2-(2,2,6,6-tetramethyl-piperidin-1-oxyl-4-ylamino)-2-oxoethyl-triphenylphosphonium chloride, Enzo Life Sciences) for 2 weeks to scavenge mitoROS; and (d) subcutaneous injection of 60 mg/kg S107 (Millipore) for 1 week. S107 is known to inhibit resting Ca^2+^ leak through the SR Ca^2+^ release channel, RyR2 (71). USP sterile water or plain liposome injection was used as a placebo control.

A group of normal C57BL/6J mice (male and female, The Jackson Laboratory) were intravenously injected with AAV9 (5 × 10^11^ genome copies/mouse) at the age of 9 weeks. The AAV9 vector (VectorBuilder Inc.) is driven by atrial natriuretic factor promoter to overexpress MCP-1 specifically in atrial CMs. The sex- and age-matched control mice were injected with the same copy number of AAV9-EGFP control virus.

All subsequent tests were performed at 36–38 weeks of age or at 1 month after virus injection.

### Echocardiographic evaluation of cardiac function.

Echocardiography was performed using the Vevo 2100 (VisualSonics) ultrasound system as in previous studies (9). Mice were anesthetized with 1%–2% isoflurane in oxygen at 1 L/min with the body temperature and the heart rate maintained at 37°C–38°C and above 400 bpm, respectively, during the scan. B-mode images along the left ventricular parasternal long axis and then M-mode images at the mid-papillary levels were obtained to calculate ejection fraction and chamber size. E/E′ was assessed in the subcostal 4-chamber view by pulsed-wave and tissue Doppler imaging to evaluate diastolic function. Measurements were averaged from 5 consecutive beats during expiration.

### Programmed intracardiac stimulation.

Programmed intracardiac stimulation was performed to assess AF inducibility as described previously (72). A standard limb ECG was recorded from subcutaneously inserted needle electrodes. Atrial and ventricular intracardiac electrograms were recorded using a 1.1 F Millar electrophysiology catheter (Millar Instruments) advanced through the right jugular vein into the right ventricle. Surface and intracardiac electrophysiology parameters were recorded at sampling rate of 4,000 Hz. Right atrial pacing was performed using 2-millisecond current pulses delivered by an external stimulator (STG2004, Multi Channel Systems) along with MCStimulus software (Multichannel System). AF was induced by an overdrive pacing protocol, starting with 2-second burst pacing at a cycle length of 40 milliseconds and decreasing in each successive burst by a 2-millisecond decrement to a cycle length of 10 milliseconds. Inducible AF was defined as the occurrence of rapid, fragmented atrial electrograms with irregular R-R intervals lasting at least 1 second. To determine whether AF inducibility was reproducible, mice were subjected to the same atrial burst-pacing protocols 3 times, and only the mice that exhibited evoked AF ≥2 times by pacing were considered AF positive. The programmed pacing was performed in a blinded manner.

### Immunoblot analysis.

Briefly, the cardiac tissue from both atria were homogenized with tissue protein extraction reagent (Thermo Fisher Scientific) and protease/phosphatase inhibitor cocktail (Thermo Fisher Scientific). Proteins were separated on SDS-PAGE gels and transferred to 0.2 μm polyvinyl difluoride membranes. After incubation with 5% nonfat milk for 1 hour at room temperature, the membranes were incubated with the corresponding primary antibodies overnight at 4°C (Cell Signaling Technology: anti-MCP-1, 2029; anti- IL-1β, 12242; anti-CD68, 97778; and anti-Vinculin, 4650; Millipore Sigma: anti–oxi-CaMKII, 07-1387; Abcam: anti-GAPDH, ab9484 and anti-CaMKII, ab52476; Badrilla: anti-RyR2 (pSer2814), A010-31; Thermo Fisher Scientific, anti-RyR2, MA3-916), followed by incubation with appropriate horseradish peroxidase–conjugated secondary antibodies for 1 hour at room temperature. Bound antibodies were visualized by chemiluminescence detection and optical density of the bands was analyzed with Image Lab Software (Bio-Rad Laboratories). The quantification data were exhibited as a ratio between target protein and housekeeping protein and normalized to the respective control.

### Quantitative real-time PCR.

Total RNA was extracted from the left and right atrial tissues utilizing the RNeasy Plus Mini Kit (Qiagen) according to the manufacturer’s instructions. The purity and concentration of the isolated RNA were assessed spectrophotometrically. Subsequent to extraction, reverse transcription was conducted to synthesize cDNA from the total RNA using the High-Capacity cDNA Reverse Transcription Kit (Thermo Fisher Scientific), following the protocol provided by the manufacturer. Quantitative PCR assays were performed to evaluate the expression levels of specific genes. These assays were carried out using the PowerUp SYBR Green Master Mix (Applied Biosystems) on a 7500Fast Real-Time PCR System (Applied Biosystems). Specific primer sets were employed to amplify target gene sequences (mouse *Ccr2* forward primer – GCTGTGTTTGCCTCTCTACCAG, reverse primer – CAAGTAGAGGCAGGATCAGGCT; mouse *Gapdh* forward primer – CTTCAACAGCAACTCCCACTCTT, reverse primer – TGTCATACCAGGAAATGAGCTTGA). The relative gene expression levels were calculated using the 2^–ΔCt^ method, with normalization to endogenous control gene expression to account for variability in cDNA input levels.

### Atrial CM isolation.

Atrial CMs were isolated as described previously (8). Briefly, hearts were excised under isoflurane (2%) anesthesia and perfused with buffer (in mM: 113 NaCl, 4.7 KCl, 0.6 Na_2_HPO_4_, 0.6 KH_2_PO_4_, 1.2 MgSO_4_, 0.032 Phenol Red, 12 NaHCO_3_, 10 KHCO_3_, 10 HEPES, 30 Taurine, 10 2–3-butanedione monoxime) for 7 minutes at a flow rate of 2.8 mL/min using a temperature controlled Langendorff perfusion system, followed by collagenase II perfusion (1.3 mg/mL, Worthington Biochemical Co.) for 10 minutes at 37°C. Both left and right atria were collected, gently cut into small pieces, and dissociated into single cells by pipetting. CMs were separated from interstitial cells by settling for 10 minutes. The cell pellet was then collected for mitoROS measurement.

### MitoSOX Red staining and mitoROS measurement.

MitoROS was measured in the isolated atrial CMs by an inverted confocal laser scanning microscope (Olympus Life Science Solutions Americas Corp.) as described previously (73). Briefly, isolated CMs were resuspended in standard Tyrode’s solution, containing (in mmol/L) 140 NaCl, 5.4 KCl, 1 MgCl_2_, 10 HEPES, 1.8 CaCl_2_, and 5.5 glucose (pH 7.4), with serially increasing Ca^2+^ concentrations (0.2, 0.5, and 1 mM), before treated with MitoSOX Red (5 μM; Thermo Fisher Scientific) for 10 minutes at 37°C in a 95%/5% O_2_/CO_2_ incubator. MitoSOX was excited by laser at 514 nm, and the emission was collected at 560 nm. Digital images were taken at 2,048 × 2,048 pixels using ×40 objective lens with <1 μm optical sections. The relative MitoSOX Red mean fluorescent intensity was obtained from the subtraction between the mean fluorescent intensity of the cells and the background of the same images with ImageJ Fiji Software, normalized to the respective control.

### Statistics.

Continuous data were presented as mean ± SEM. For the dot plots, the lines indicated the mean values, and the error bars indicated SEM. Data were analyzed using a 2-tailed Student’s *t* test or 1-way ANOVA with Bonferroni’s post hoc tests for multiple pairwise comparisons. Categorical data were compared using Fisher’s exact test. All statistical analyses were performed with GraphPad Prism 5.0. A *P* value of less than 0.05 was considered statistically significant.

### Study approval.

Animal care and interventions were provided in accordance with the NIH *Guide for the Care and Use of Laboratory Animals* (National Academies Press, 2011), and all animal protocols were approved by the Institutional Animal Care and Use Committee of the University of Minnesota.

### Data availability.

Values for all data points in the graphs can be found in the Supporting Data Values file.

## Author contributions

XZ designed the study; conducted most of the animal experiments, part of the immunoblot tests,and mitoROS measurements on control and DM CMs; analyzed data; interpreted results; and wrote the manuscript. HL conducted glucose tests, AAV9 vector injection, echocardiography, programmed intracardiac stimulation on MCP-1–overexpressing mice, CM isolation, and part of the immunoblot tests; analyzed data; interpreted results; and wrote the main body of the manuscript. Because XZ designed, initiated, and obtained the data for the original experiments, she was listed first; however, the amount of work done by XZ and HL for this manuscript was ultimately equivalent. Both authors agreed to this author order. GJK conducted AAV9 vector design and qRT-PCR assay. ML measured mitoROS on MCP-1–overexpressing and control CMs. FF conducted CMs isolation and intravenous injection of AAV9 vectors. YG conducted postsurgical animal care and assisted with immunoblotting. SCD designed and supervised the study, interpreted results, provided project resources, and wrote the manuscript. All authors reviewed and commented on the manuscript.

## Supplementary Material

Supplemental data

Supporting data values

## Version 1 06/18/2024

In-Press Preview

## Version 2 08/08/2024

Electronic publication
