# Supplementary material for: Macrophage IL-1β mediates atrial fibrillation risk in diabetic mice
Source: JCI Insight. 2024 Jun 18;9(15):e171102. doi: 10.1172/jci.insight.171102 (PMC11383594; doi:10.1172/jci.insight.171102)

SUPPLEMENTARY MATERIALS

Macrophage interleukin-1β mediates atrial fibrillation risk in diabetic mice

SUPPLEMENTAL METHODS

Cryosection and Masson's trichrome staining:

Excised atria were embedded in Tissue-Tek O.C.T. compound (Sakura Finetek USA, Inc., Torrance, CA) and immediately frozen in liquid nitrogen. Embedded atria were then cut into 8 µm-thick sections with a Leica cryostat. Collagen staining was performed using the Trichrome Stain Kit (Abcam, #AB150686, Boston, MA) according to the manufacturer's protocol.

Immunofluorescence staining

Macrophage infiltration was examined by immunofluorescence staining. Atria cryosections were fixed in ice-cold acetone for 10 minutes. After washing 3 times with PBS for 5 minutes each, sections were blocked with 3% BSA/0.3% TritonX-100/5% goat normal serum/PBS for 1 hour at room temperature and then incubated with the 1:300 diluted primary antibody in 1% BSA/PBS (Cell Signaling Technology, anti-CD68, #97778, Danvers, MA) overnight at 4°C, followed by 1:500 diluted Alexa Fluor 488–conjugated secondary antibody (Thermofisher Scientific, goat-anti-rabbit IgG, #A-11008, Waltham, MA) for 1 hour at room temperature. Sections were then mounted with Vectashield DAPI-containing fluorescence-mounting medium (Vector Laboratories, #H-1200, Newark, CA) and imaged with a Zeiss upright microscope.

.

Supplemental Table 1. Echocardiographic characterization of control and diabetic mice.

	Ctrl (N=6)	DM (N=6)	P value
HR (bpm)	505 ± 18	491 ± 20	NS
LVEDV (μL)	75 ± 3	72 ± 3	NS
LVESV (μL)	37 ± 2	34 ± 3	NS
EF (%)	50 ± 1	53 ± 3	NS
E/E'	13.6 ± 1.0	23.2 ± 1.7	<0.001
LA dimension (mm)	2.0 ± 0.1	2.1 ± 0.1	NS

Note: Data are means ± SEM; Unpaired t-test was used. Ctrl, control; DM, diabetes mellitus; EF, ejection fraction; E/E', ratio of transmitral Doppler early filling velocity to tissue Doppler early diastolic mitral annual velocity; HR, heart rate; LA, left atrium; LVEDV, left ventricular end-diastolic volume; LVESV, left ventricular end-systolic volume; NS, no significance.

Supplemental Table 2. Echocardiographic characterization of MCP-1 overexpressing mice

	AAV9-plain (N=9)	AAV9-MCP-1 (N=9)	P value
HR (bpm)	507 ± 11	484 ± 10	NS
LVEDV (μL)	67 ± 5	64 ± 2	NS
LVESV (μL)	31 ± 3	31 ± 2	NS
EF (%)	55 ± 2	52 ± 2	NS
E/E'	16.5 ± 0.6	15.3 ± 1.6	NS
LA dimension (mm)	1.9 ± 0.1	2.3 ± 0.1	0.023

Note: Data are means ± SEM; Unpaired t-test was used. AAV9, atrial-specific adeno-associated viral vectors (serotype 9); EF, ejection fraction; E/E', ratio of transmitral Doppler early filling velocity to tissue Doppler early diastolic mitral annual velocity; HR, heart rate; LA, left atrium; LVEDV, left ventricular end-diastolic volume; LVESV, left ventricular end-systolic volume; MCP-1, monocyte chemoattractant protein-1; NS, no significance.

Supplemental Figure 1. Representative Masson's trichrome staining of atria. No apparent atrial fibrosis was detected in control or diabetic mice. Scale bar represents 100μm. Ctrl, control; DM, diabetes mellitus.

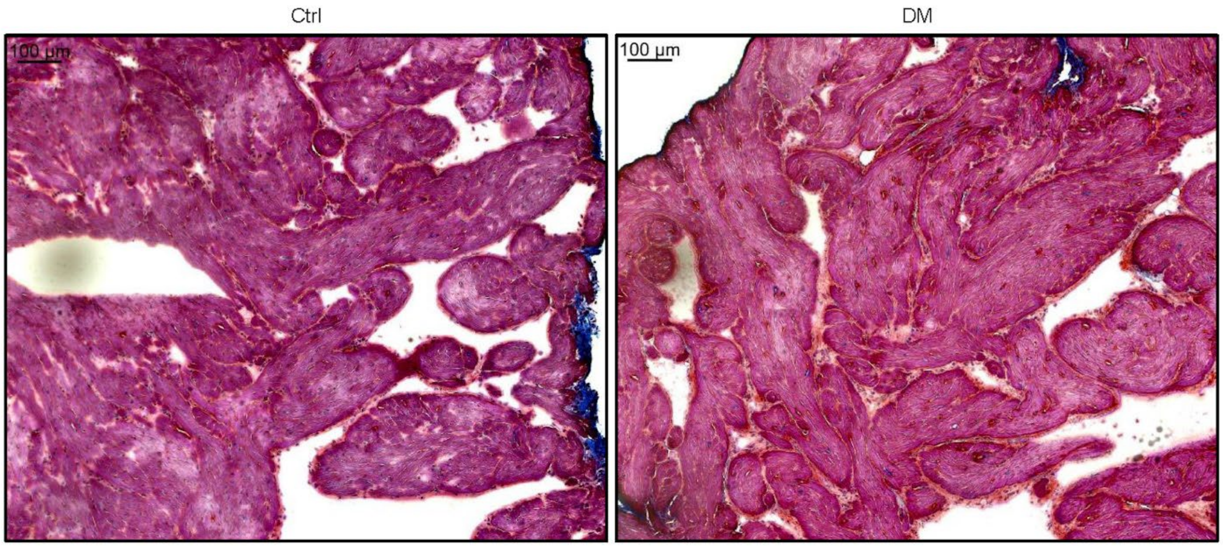

Supplemental Figure 2. Representative immunofluorescence staining of cardiac macrophages in control and diabetic atria. Macrophage was identified by CD68 staining. Nuclei were stained with DAPI. Scale bar represents 100μm. Ctrl, control; DM, diabetes.

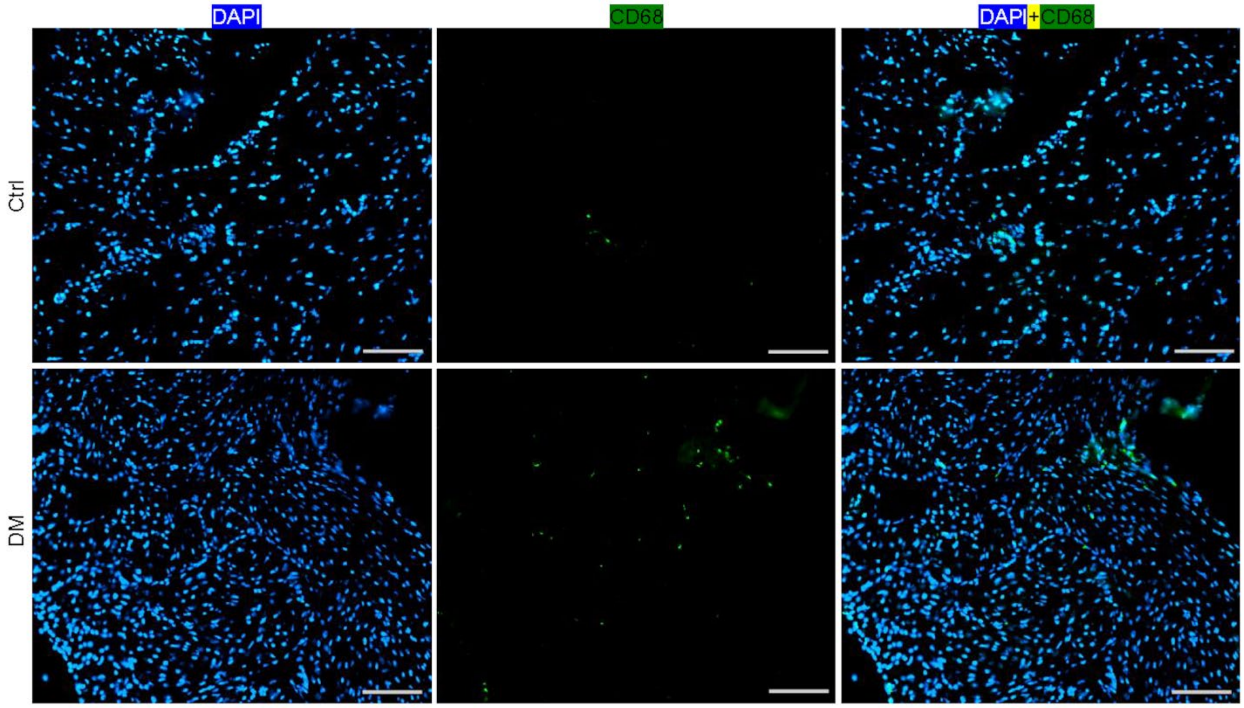

Supplement: Supplemental data [file jciinsight-9-171102-s255.pdf]
